# An Explainable Artificial Intelligence Software Tool for Weight Management Experts (PRIMO): Mixed Methods Study

**DOI:** 10.2196/42047

**Published:** 2023-09-06

**Authors:** Glenn J Fernandes, Arthur Choi, Jacob Michael Schauer, Angela F Pfammatter, Bonnie J Spring, Adnan Darwiche, Nabil I Alshurafa

**Affiliations:** 1 Department of Computer Science Northwestern University Evanston, IL United States; 2 Department of Preventive Medicine, Feinberg School of Medicine Northwestern University Chicago, IL United States; 3 Department of Computer Science Kennesaw State University Kennesaw, GA United States; 4 Department of Computer Science University of California, Los Angeles Los Angeles, CA United States

**Keywords:** explainable artificial intelligence, explainable AI, machine learning, ML, interpretable ML, random forest, decision-making, weight loss prediction, mobile phone

## Abstract

**Background:**

Predicting the likelihood of success of weight loss interventions using machine learning (ML) models may enhance intervention effectiveness by enabling timely and dynamic modification of intervention components for nonresponders to treatment. However, a lack of *understanding* and *trust* in these ML models impacts adoption among weight management experts. Recent advances in the field of explainable artificial intelligence enable the interpretation of ML models, yet it is unknown whether they enhance model understanding, trust, and adoption among weight management experts.

**Objective:**

This study aimed to build and evaluate an ML model that can predict 6-month weight loss success (ie, ≥7% weight loss) from 5 engagement and diet-related features collected over the initial 2 weeks of an intervention, to assess whether providing ML-based explanations increases weight management experts’ agreement with ML model predictions, and to inform factors that influence the understanding and trust of ML models to advance explainability in early prediction of weight loss among weight management experts.

**Methods:**

We trained an ML model using the random forest (RF) algorithm and data from a 6-month weight loss intervention (N=419). We leveraged findings from existing explainability metrics to develop Prime Implicant Maintenance of Outcome (PRIMO), an interactive tool to understand predictions made by the RF model. We asked 14 weight management experts to predict hypothetical participants’ weight loss success before and after using PRIMO. We compared PRIMO with 2 other explainability methods, one based on feature ranking and the other based on conditional probability. We used generalized linear mixed-effects models to evaluate participants’ agreement with ML predictions and conducted likelihood ratio tests to examine the relationship between explainability methods and outcomes for nested models. We conducted guided interviews and thematic analysis to study the impact of our tool on experts’ understanding and trust in the model.

**Results:**

Our RF model had 81% accuracy in the early prediction of weight loss success. Weight management experts were significantly more likely to agree with the model when using PRIMO (χ^2^=7.9; *P*=.02) compared with the other 2 methods with odds ratios of 2.52 (95% CI 0.91-7.69) and 3.95 (95% CI 1.50-11.76). From our study, we inferred that our software not only influenced experts’ understanding and trust but also impacted decision-making. Several themes were identified through interviews: preference for multiple explanation types, need to visualize uncertainty in explanations provided by PRIMO, and need for model performance metrics on similar participant test instances.

**Conclusions:**

Our results show the potential for weight management experts to agree with the ML-based early prediction of success in weight loss treatment programs, enabling timely and dynamic modification of intervention components to enhance intervention effectiveness. Our findings provide methods for advancing the understandability and trust of ML models among weight management experts.

## Introduction

### Background

Obesity-attributable medical costs remain high for health care systems and patients and are on the rise [1,2]. Although there has been an increase in the number of mobile intervention‒based weight loss treatment programs [3-5], effective and economical interventions have not been adequately identified or disseminated. To save costs and improve health outcomes, interventions that can predict nonresponse early during a treatment program enable dynamic modification of intervention components (ie, stepped-care models [6]) to steer the course of treatment toward achieving a response or the desired health outcome, analogous to clinical decision-making over time. Clinical decision-making involves making a decision that maximizes what the patient values, and so, treatment decisions could be informed by better predictions of what treatments work for whom and when. Machine learning (ML) models help make early predictions; however, building models to predict human behavior early on (eg, if a patient will lose weight in the near future) poses several challenges [7-10].

A growing body of literature provides evidence for using ML models to understand health behavior [11-14]; however, 3 critical challenges are associated with using ML models to predict health behavior. The first is that ML models used to represent complex health behavior data are often black boxes that are overly complex; for example, a random forest (RF) algorithm—a classifier known for increasing predictive accuracy even without hyperparameter tuning, comprising many decision trees where each tree uses a different number of features or variables to determine a classification—is difficult to understand intuitively and, by extension, difficult to use in practice. Although RF outperforms several other ML methods in prediction accuracy, it is notoriously hard to interpret [15]. There is an apparent trade-off between the performance of classifiers (accuracy) and their ability to explain the reasoning behind their results (explainability). Despite this, researchers have been quick to use the predictability waves of RF algorithms. Several publications have used RF algorithms for critical tasks such as risk prediction [16,17], estimating energy prediction [18], and early detection of depression [19]; however, their clinical utility has yet to be realized, which may be because of the lack in using explainability.

Explainability methods developed by computer scientists might help users gain insight into the inner workings of a model. However, it is unknown whether weight management experts will agree with predictions provided by ML models even when visualization and interactive design components included in these techniques attempt to convey to domain experts the reasoning behind the decisions made by the model [20,21]. Research has highlighted an overreliance problem among clinicians in primary care settings, where the misuse of a system can occur when placing too much trust in automated systems, resulting in user agreement with incorrect system suggestions [22]. However, Jacobs et al [23] recently reported that psychiatrists with higher familiarity with ML were less likely to use an ML recommendation of which antidepressant drug to use compared with clinicians with lower ML familiarity. However, overreliance can be mitigated by using explainability metrics. This leads to the second challenge: health behavior is often more difficult to predict, so it is unknown whether there would be a lack of adoption of ML or overreliance. Consequently, it remains unknown whether weight management experts are less likely to use ML recommendations even when attempting to increase credibility with explainability metrics.

Traditional ML evaluation metrics, such as accuracy and sensitivity, provide an overall metric of the model’s performance but fall short of providing insights into the reasoning for the model’s prediction. Recent developments in the field of explainable artificial intelligence (AI) have resulted in new explainability metrics or explanations that provide a further understanding of the reasoning behind the decisions made by an ML model. One example of an explainability metric, or explanation, is feature ranking to understand the degree to which features influence a model. Model-agnostic explanations, such as Shapley additive explanations (SHAP) [24], local interpretable model-agnostic explanations (LIME) [25], and Anchors [26], are currently the standard approaches to explainability. LIME and Anchors rely on local explanations that aim to explain the model’s reasoning for a given instance (eg, to answer the question, Why was this instance classified as *X*?); however, these algorithms are based on approximations by defining the contribution of a feature to the difference between the actual and mean prediction (ie, inaccurate explanations). Although local explanations are specific to understanding a specific instance, when combined with explanations of multiple instances, they could provide an understanding of the model’s overall behavior, similar to a global explanation. Global explanations describe the overall working of the ML model [27], such as feature importance. For example, a local explanation would be generating explanations for a prediction made by an ML model trained to predict whether a patient would lose weight. However, a global explanation would be looking at feature importance to understand which features contributed more toward the model’s capability to discern one class from the other.

SHAP by Lundberg and Lee [24] can create global explanations by aggregating Shapley values (an approach with a solid foundation in game theory) to create feature importance, summary, and dependence plots. Shapley values are feature attributions that act as driving forces, either contributing to the prediction or not contributing to the prediction. This implies that, unlike LIME, SHAP does not train an interpretable model that can make predictions. The literature has since defined a set of principles for designing explanations.

The use of prime implicants as explanations for weight management experts is supported by current literature describing the design principles for human explanations [28]. These principles state that explanations should be “contrastive,” explaining why the model predicted an instance as one class over another. A prime implicate explanation denotes a region that is sufficient to arrive at a given prediction. To arrive at a different prediction, it is necessary (but not sufficient) to go outside that region. Thus, prime implicants also follow the second principle of being exhaustive.

We designed Prime Implicant Maintenance of Outcome (PRIMO; our interactive explainable software) to translate prime implicants into understandable quantities (Multimedia Appendix 1). The explanation’s primality leads it to be parsimonious. The authors additionally emphasize the need to provide guidelines for the effective use of interactive explanation tools. Our explanation tool provides users with a step-by-step intuitive approach for generating and evaluating explanations. Our interactive explainability tool, PRIMO, is designed based on these design principles in addition to leveraging methods from SHAP, LIME, and Anchors.

The need for exact, accurate explanations that health experts can intuitively visualize and interact with led to our unique approach of extending the methods developed by Shih et al [29-31] by compiling an RF classifier into ordered decision graphs, a tractable representation also called ordered binary decision diagrams (OBDDs). We used this tractable representation alongside visualization methods used in prior research to build PRIMO, a tool to generate, visualize, and interact with prime implicant‒based explanations. However, the third challenge arises despite prior research among other domain experts; it is unknown whether explainability methods advance the trust and understanding of ML models among weight management experts. The primary objective of this case study was to build a weight loss prediction model to assess whether providing ML-based predictions and explanations from this model increases weight management experts’ agreement with ML model predictions of success or failure and to conduct evaluation studies with weight management experts to understand the impact of such models on the trust and understanding of ML models and ultimately adoption in the real world. This work aimed to support the use of explainable ML to support decisions related to changing the course of treatment for someone who has already started treatment based on their initial response.

### Explainability Definition

Several explainability AI methods have been designed to improve the interpretability of ML models. Kim et al [32] tested an interpretability method designed for conditional recurrent neural networks to predict weight loss at 16 weeks using features collected across the 16-week study. However, this method does not apply to RF models and does not focus on early weight loss prediction. Explainability AI involves the communication of ML model results and operations for different audiences and purposes, and our main goal is to study its effectiveness in communicating these explanations to the weight management expert audience. To do this, we defined explainability as the ability of weight management experts to trust and understand the explanations presented when related to the problem at hand, predicting weight loss.

## Methods

### Overview

**Figure 1:**
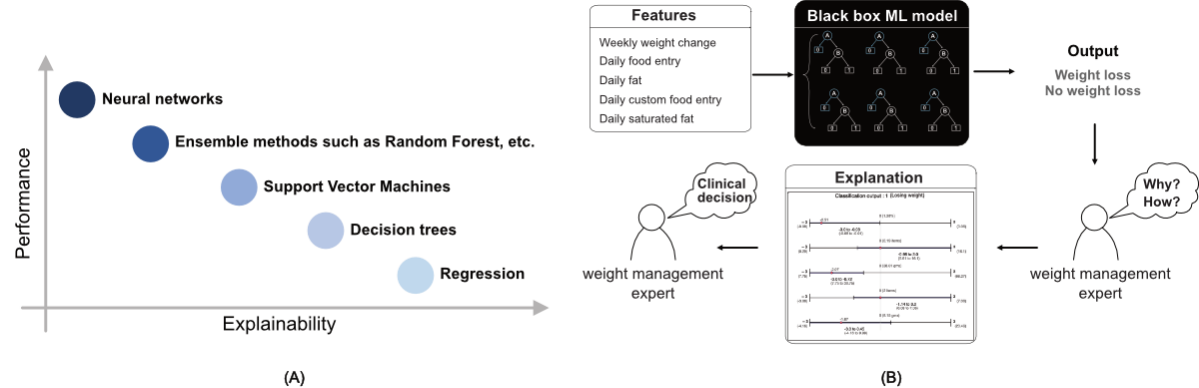
(A) Trade-off between the performance of a model (accuracy) and the explainability. (B) Black box machine learning (ML) model.

Figure 1 shows our proposed framework in three steps: (1) selecting an optimal early prediction time point, (2) generating a tractable decision diagram representation of the RF model using the RF classifier, and (3) building a software tool to enable visualization and interaction with the explanations. We have described each of these steps in the subsequent sections, followed by details of our one-on-one interviews with domain experts using our software tool, the statistical analysis to assess the agreement of weight management experts with our ML model output, and the questions used to assess the trust and understanding of ML models.

### Weight Loss Study

The Opt-IN study was a 6-month, theory-guided, and technology-supported weight loss intervention to explore the factors contributing to substantial weight loss. The details of the study have been described previously [33,34]. This study enrolled adults with a BMI between 25.1 kg/m^2^ and 39.9 kg/m^2^. Eligible participants had stable weight (no loss or gain >11.3 kg for the past 6 months), were not enrolled in any formal weight loss program or taking weight-reducing medications, and were interested in losing weight. The participants obtained their personal physician’s approval to participate, and the physician agreed to receive the study reports. All study procedures were approved by an institutional review board, and all participants provided written informed consent before enrollment. We used demographic information and data collected from participants’ smartphones to determine what factors early in the intervention predicted weight loss at 6 months. We defined clinically meaningful weight loss as ≥7% weight reduction from baseline. In the Diabetes Prevention Program, 7% weight loss over 6 months led to a 58% reduction in the development of diabetes [35].

### Step 1: Optimal Early Prediction Time Point and Building an RF Model for Early Prediction

To identify the critical early time point for building a machine learned model, we built several learned models at different time points to select the model‒time point pair with the highest predictability. We combined both evidence-based and data-driven practices to guide the process. Evidence-based practice guided our initial selection of features and the subset of time points for early prediction to select from [36-42]. Data-driven approaches guided the dimensionality reduction through feature selection and the development of a machine learned model at each time point. We built multiple models using the RF classifier to predict a binary weight loss outcome at the end of 26 weeks. We observed a local optimum in the predictability of models at the end of weeks 2 and 3; we decided to select an earlier time point and therefore selected the end of week 2 as the optimum time point. We used potential predictors to build an RF classifier observing the change over time in data collected from baseline until the end of week 2 to predict a weight loss outcome at the 6-month time point [37,38].

On the basis of RF feature importance analysis and prior literature supporting the potential for initial weight loss [36-38], fat intake [39,40], and patterns of engagement with mobile health tools to predict health behavior trends [41,42], we identified a minimal number of highly predictive features combining self-reported dietary variables (initial weight loss, fat intake, and saturated fat intake) and engagement variables (entry of food items and entry of custom food items) to build the RF model. This enabled the reduction of the number of features from 10 features (Multimedia Appendix 2) to 5 features, resulting in reduced complexity and further aiding the understanding of generated explanations. After rejecting participants with missing data points, we trained the RF classifier (N=419) using these 5 features in the *scikit-learn* Python library and optimized the classifier to have 13 trees, a maximum depth of 3 for each tree, and an accuracy of 81%.

### Step 2: Generating a Tractable Representation to Facilitate the Computation of Prime Implicant Explanations

**Figure 2:**
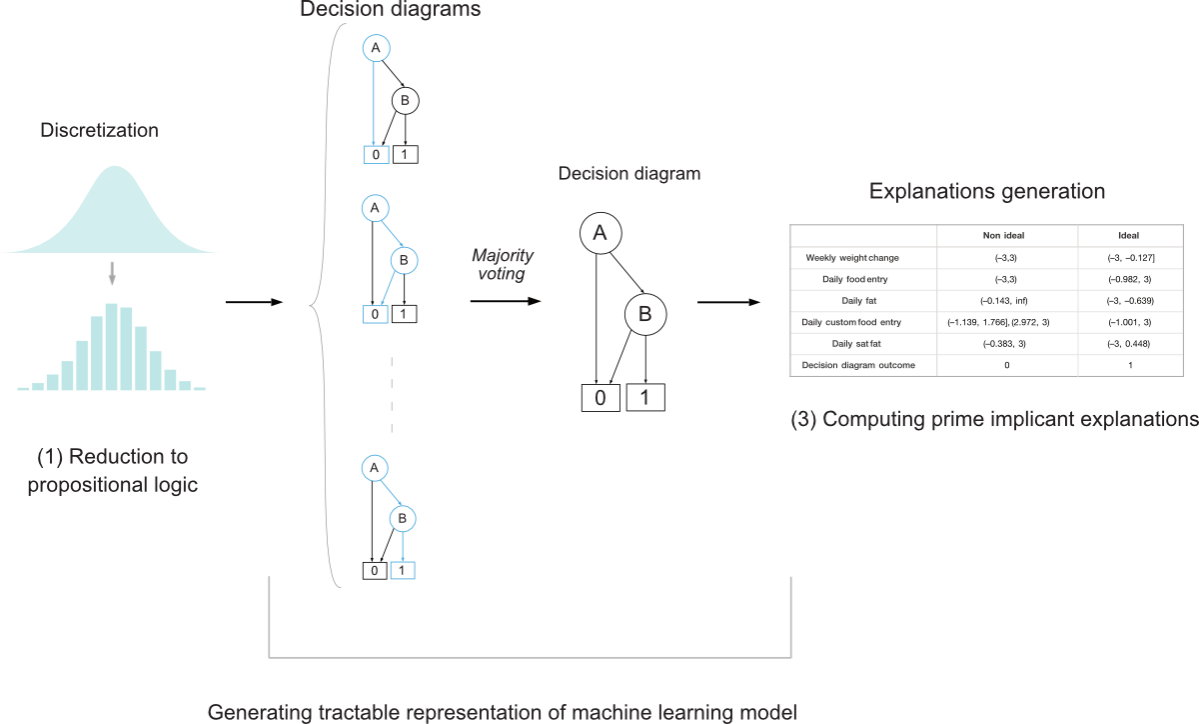
Generating tractable representation of machine learning model to compute prime implicant explanations.

The RF model underwent 3 steps (Figure 2).

#### Reduction to Propositional Logic

An RF classifier generally takes continuous variables as input, but each continuous feature becomes a proposition when it appears in any given decision tree. Hence, we can view any RF classifier’s input or output behavior as a propositional function. An RF classifier consists of an ensemble of decision trees and classifies an instance by evaluating each decision tree. Note that an RF classifier (and each decision tree in its ensemble) evaluates instances using various tests of the form *x_i_*≥*t* or *x_i_*<*t*, both propositions. When we take all the variable tests for a particular feature *X_i_*, they induce a partitioning of the space of *X_i_* into mutually exclusive and exhaustive intervals. In our reduction, we represent each interval as a binary variable in our propositional formula, which can be viewed as a discretization of the original continuous variable.

We defined a propositional formula for the RF by defining a propositional variable for each interval appearing in the RF. Given a leaf node in a decision tree, the propositional formula for the leaf node’s class label is the conjunction of the decisions found on the path from the root to the leaf. Given a decision tree, the propositional formula for a class label is the disjunction of the paths’ formulas to each class label leaf. The propositional formula of a random for a class label is determined by aggregating the formulas for each decision tree using a majority gate. For a given assignment of features to values, the resulting propositional formula is true if the RF labels the feature vector with the corresponding label.

#### Generating Tractable Representation (Conversion to OBDD)

Simply obtaining the propositional formula underlying an RF is not helpful because reasoning with the formula will not be tractable. For example, testing whether there exists a satisfying assignment (ie, testing whether there exists some feature vector that obtains a given label) is a nondeterministic polynomial time hard problem. Hence, we appeal to the field of *knowledge compilation* to obtain a tractable representation of the formula [43]. Knowledge compilation is a subfield of AI that studies, in part, tractable Boolean circuits and the trade-offs between succinctness and tractability, that is, by enforcing different properties on the structure of a Boolean circuit, one can obtain greater tractability (the ability to perform specific queries and transformations in polytime) at the possible expense of succinctness (the size of the resulting circuit). We followed Choi et al [31] to compile the propositional formula of an RF into an ordered decision graph or, equivalently, an OBDD. We adapted an RF compiler [44] to compile a propositional formula into an OBDD; many queries of interest become tractable, typically requiring time that is only linear in the size of the resulting OBDD.

#### Computing Prime Implicant Explanations

To generate explanations, we compiled the discrete RF classifier into an OBDD, which is a tractable function representation and can be used to answer queries and facilitate efficient explanations of classifiers. Once we have an OBDD representation of our RF classifier, we reason about and generate explanations for the behavior of the classifier [45,46]. One type of explanation is called a *sufficient explanation* [47], which corresponds to computing the prime implicants of an RF classifier’s propositional function. A prime implicant of an RF’s propositional formula can be considered as a minimal assignment of features to values that will fix the output of the RF classifier. It is a partial feature vector *sufficient* to fix the classifier’s decision. The behavior of an RF can be wholly described as a disjunction of all its prime implicants (also called its prime cover). Given a propositional formula represented as an OBDD, efficient algorithms exist for computing its prime implicants [48,49].

**Figure 3:**
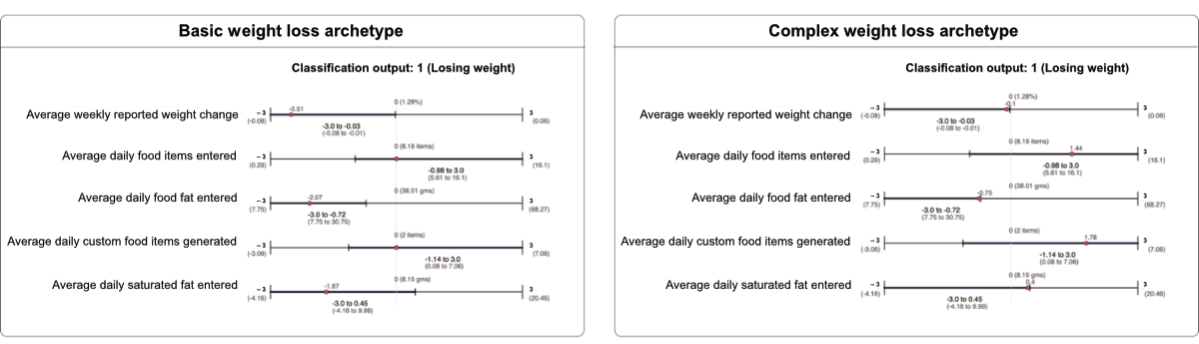
In the 2 Prime Implicant Maintenance of Outcome (PRIMO) visuals, the red point indicates the patient profile for basic and complex weight loss archetypes. The complex weight loss archetype tends to be difficult because the red points fall at the edges of the PRIMO-generated prime implicant ranges.

For each instance that is input into the classifier, we generated a shortest prime implicant that is compatible with it (ie, the shortest subinstance that is also a prime implicant). As explained by Shih et al [30], one way to verify the behavior of a classifier is to verify whether the classifier is compatible with the expectations of a domain expert. A domain expert may define the expectations of input-output pairs for a classifier to be reliable. For example, a domain expert may say that anyone who has lost ≥1% of their weight and is maintaining low intakes of saturated fat early in treatment is on the trajectory toward clinically significant distal weight loss. To facilitate this understanding of expected behavior, we provide a visual of the intervals or ranges for each variable, such that if the value of each variable falls within these ranges, the classifier output will remain the same. Figure 3 shows 2 sample visuals of a basic weight loss archetype and a complex weight loss archetype along with their corresponding visuals, which we call PRIMO.

### Step 3: Designing a Tool for Weight Management Experts

**Figure 4:**
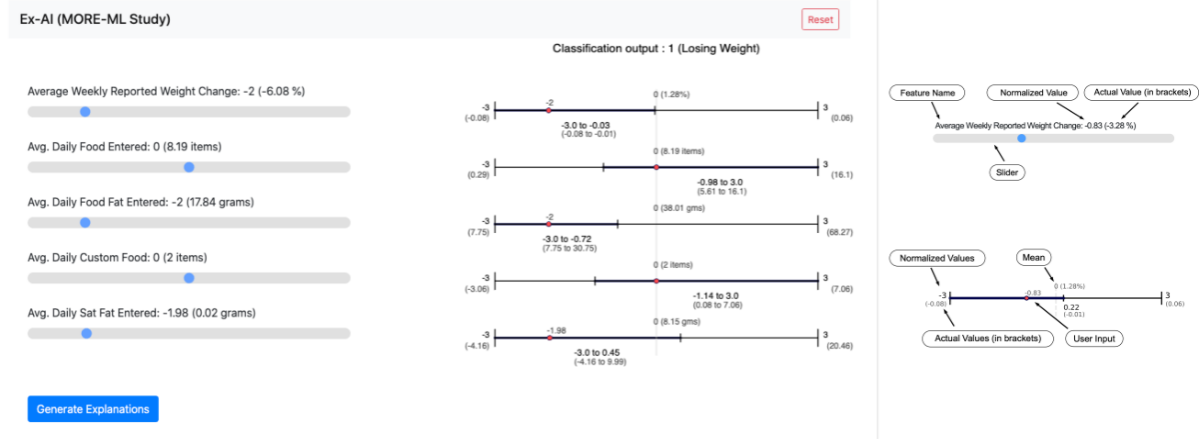
Prime Implicant Maintenance of Outcome software interface. Ex-AI: explainability artificial intelligence; ML: machine learning.

An interactive software tool (Figure 4) was designed to enable researchers to query the ML model and generate explanations for the hypothetical archetypes of individuals. The user creates custom instances by assigning values to each feature and selecting a “Generate Explanation” button. The software queries the interpretable model to provide a prediction and PRIMO’s visual. PRIMO was designed to be interactive and intuitive by enabling users to enter values for model features by adjusting sliders that represent standardized values for each feature. The slides show the *z* score and corresponding actual value for each feature to create an instance.

The explanation provided by the model is displayed in the form of highlighted ranges on the interface. If one were to tweak the input values of an instance within the respective ranges specified by the explanation, the model’s prediction and explanation are guaranteed not to change. However, on tweaking the input value outside the displayed ranges, the prediction and explanation may or may not change. This allows users to understand the model’s limits that may or may not produce a different output, thereby providing better insight into the model’s functionality. In some cases, there is a possibility that, on selecting specific inputs, there is no explanation range for one or more features. This indicates that given the values for all the features with defined ranges, the value of the feature without a range does not affect the output of the predictor.

### Evaluation Study With Weight Management Experts

**Figure 5:**
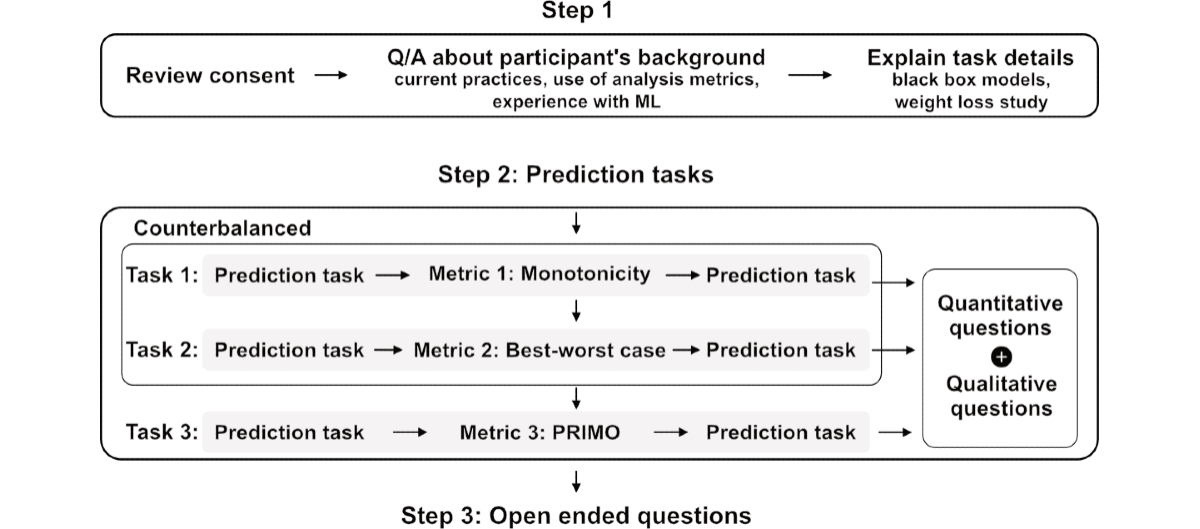
Study design. ML: machine learning; PRIMO: Prime Implicant Maintenance of Outcome.

A total of 14 participants (Multimedia Appendix 3) were recruited (mean age 45, SD 5 years) for one-on-one interviews conducted over an interactive videoconference. The length of each interview was approximately between 1 and 1.5 hours. The participants included mobile health researchers for weight loss. Participants were recruited from an obesity research special interest group mailing list and listservs focusing on weight loss research. Participants had backgrounds in ≥1 of the following areas: psychology, nutrition, epidemiology and clinical experience, statistics, and data science. The study’s primary goal was to evaluate among end users, the weight management experts, whether the explanations are understandable and whether they would trust the tool for use in a real-world scenario. Figure 5 shows the overall study design.

Participants were asked about their background and experience with analysis metrics and ML. They were presented with details of the weight loss intervention for which the RF model was based. They were then required to provide feedback on 2 other explanation types besides PRIMO. These 2 explanation types were used to help participants become acquainted with the idea of explainability and to use explainability tools to understand predictions from an ML classifier.

**Figure 6:**
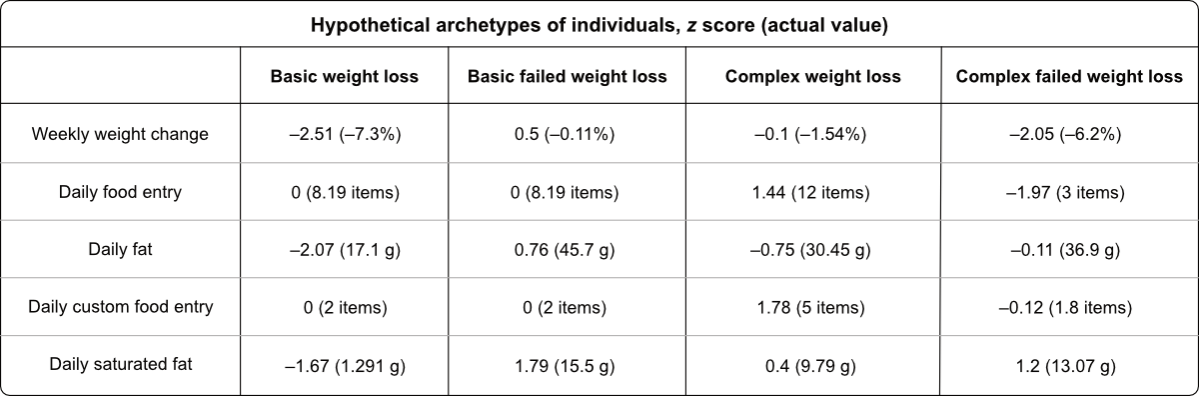
Hypothetical archetypes shown to participant types. Values presented as z score (actual value).

Our explainability framework enabled the generation of the other 2 explanation types (Multimedia Appendix 4). (1) Monotonicity (baseline metric 1), which closely resembles feature ranking, was where we calculated for each feature a percentage that denotes the proportion of instances for which the classifier was either monotonically increasing or decreasing. For example, we would expect that the greater the reported weight reduction, the more likely the participant was to lose weight; if this were always the case, it would be 100%. (2) Best-worst case (baseline metric 2), which closely resembled a conditional probability approach, was designed to convey the information gained about the class label of an instance based on observing a particular feature and value pair. In the best-worst case, we computed for a feature variable *X*, with value *x*, the proportion of feature vectors classified as either positive or negative class. The goal of adding these metrics was to get participants to think beyond the existing explainability metrics provided. For each metric, we created four hypothetical archetypes of individuals (Figure 6; Table 1 reports *z* scores and actual values), two basic and two complex cases for prediction, including (1) a participant expected to lose sufficient weight at the end of the study (basic weight loss), (2) a participant expected not to lose weight (basic failed weight loss), (3) a participant that was borderline but expected to lose sufficient weight (complex weight loss), and (4) a participant that was borderline and not expected to lose weight (complex failed weight loss).

We asked the participants to predict the outcome of each hypothetical archetype before and after viewing each explanation type.

**Table 1:** Hypothetical archetypes shown to participant types.

	Basic weight loss, *z* score (actual value)	Basic failed weight loss, *z* score (actual value)	Complex weight loss, *z* score (actual value)	Complex failed weight loss, *z* score (actual value)
Weekly weight change	−2.51 (−7.3%)	0.5 (−0.11%)	−0.1 (−1.54%)	−2.05 (−6.2%)
Daily food entry	0 (8.19 items)	0 (8.19 items)	1.44 (12 items)	−1.97 (3 items)
Daily fat	−2.07 (17.1 g)	0.76 (45.7 g)	−0.75 (30.45 g)	−0.11 (36.9 g)
Daily custom food entry	0 (2 items)	0 (2 items)	1.78 (5 items)	−0.12 (1.8 items)
Daily saturated fat	−1.67 (1.291 g)	1.79 (15.5 g)	0.4 (9.79 g)	1.2 (13.07 g)

### Ethical Considerations

The study was deemed exempt from ethics approval by Northwestern University’s institutional review board (STU00213879), and all weight management experts provided written informed consent before participating in the study.

### Statistical Analysis to Assess Likelihood of Agreement With ML Predictions

We used generalized linear mixed-effects models (GLMMs) to evaluate participants’ agreement with ML predictions. Our outcomes were binary (agreement yes or no), so our models used logit link functions and random participant effects to account for the correlation between outcomes within the same participant. The primary analysis evaluated the likelihood of final agreement across predictions or explainability methods. We further examined the relationships between explainability methods and two participant behaviors: (1) switching initial incorrect predictions to agree with the ML models and (2) switching initially correct predictions to disagree with the ML models. For (1), the analyses considered only the initially incorrect predictions by the participants, whereas models for (2) included only the initially correct predictions by participants. The models were fit using the *lme4* library in the R programming language (version 4.1.2; R Foundation for Statistical Computing). We fit a model with fixed explainability method effects for each of these analyses and reported the estimated effects, SEs, and 95% CIs. To examine the overall relationship between explainability methods and outcomes, we also fit a model omitting the explainability method effects and conducted likelihood ratio tests (LRTs) for nested models (Cronbach α=.05).

### Advancing Explainability Through Trust and Understanding

To further determine whether PRIMO advanced trust and understanding compared with the other baseline methods, we administered an intelligibility questionnaire and then requested the experts to rank each of the 3 explanation types and then probed them to discuss why they preferred one method over the other. Quantitative responses were provided using REDCap (Research Electronic Data Capture; Vanderbilt University), and themes were identified based on the participants’ responses to open-ended questions.

The intelligibility questions (Textbox 1 [50]) were evaluated on a 5-point Likert response scale, ranging from 0 (not at all), 1 (a little), 2 (somewhat), 3 (quite a lot), to 4 (extremely).

**Intelligibility questionnaire (the 5 quantitative questions were presented as adapted from the study by Cahour et al).**

**Trust**

- Trust: How much do you trust the model?
- Reliable: How reliable do you think the explainability metric is?

**Understand**

- Predictable: According to you, how predictable are the outputs of this model?
- Efficient: According to you, how efficient is the explainability metric in describing why the model generated the outputs or predictions?
- Confident: How confident are you in your answers?

We then calculated and compared the mean (SE) of the Likert response scale values answered by participants for the 5 quantitative questions shown after each task. At the end of the study, the participants were asked to rank the 3 explanation types and to describe their suggestions regarding improvements and which types enhanced their understanding of the ML models. We also reported on themes regarding participant preference for a specific metric type over another by qualitatively analyzing their responses.

Qualitative questions were designed to engage the user in an open-ended response to capture how and why the metric facilitates the prediction of outcomes. The 3 open-ended questions were as follows (with the first 2 adapted from the study by Ribeiro et al [25]):

- Trust: Would you trust this model guided by this evaluation metric to work well in the real world?
- Understanding: How do you think the model is able to distinguish between the classes?
- Understanding: Do you have a better understanding of the model? If yes, why? If not, how would you improve the explainability metric?

## Results

### Overview

We trained an RF model using 5 engagement and diet-related features, captured during the first 2 weeks of treatment, to predict early on at week 2 if a participant would lose weight at the end of the 6-month weight loss study. The model was trained with 81% accuracy (specificity 86% and sensitivity 69%). The five features obtained using a smartphone app were (1) average daily food entry, (2) average daily custom food entry, (3) average weekly weight change, (4) average daily fat, and (5) average daily saturated fat.

### Quantitative Findings

The GLMM results are presented in Table 2. Coefficients and 95% CIs are reported on the odds ratio scale. Across the explainability methods, participants’ initial predictions were often correct (PRIMO software, 67.9%; monotonicity, 69.6%; and best-worst case, 66.1%). Explainability methods were statistically significantly related to the final agreement (LRT χ^2^=7.9; *P*=.02), and the PRIMO software method appeared to have the strongest final agreement at 89.3% relative to the monotonicity (76.8%) and best-worst case (67.9%) methods. Explainability methods were also statistically significantly related to participants switching initially correct predictions to disagree with the ML models (LRT χ^2^=6.85; *P*=.03). Participants responding to the PRIMO software method were considerably less likely to change their initially correct responses (2.6%) relative to monotonicity (5.1%) or the best-worst case (18.9%). Finally, explainability methods were related to participants switching to initially incorrect predictions to agree with ML models (LRT χ^2^=6.27; *P*=.04). The PRIMO software method saw a greater proportion of participants correcting incorrect predictions (72.2%) relative to monotonicity (35.3%) or the best-worst case (42.1%). Notably, the between-subject variability was minimal (intraclass correlation coefficient <0.01) for a final agreement and switching to disagreement but was substantial for switching to incorrect predictions to agree with ML models (estimated between-subject SD 0.855 on the logit scale; intraclass correlation coefficient=0.18).

**Table 2:** Generalized linear mixed-effects model results.

Dependent variable	Intercept, odds ratio (95% CI)	*P* value	Monotonicity, odds ratio (95% CI)	*P* value	PRIMO^a^ software, odds ratio (95% CI)	*P* value
Final agreement (n=168 responses)	2.11 (1.22-3.90)	.01	1.57 (0.68-3.68)	0.29	3.95 (1.49-11.8)	.009
Switch to disagree (n=114 responses)	0.23 (0.071-0.501)	.001	0.23 (0.03-1.04)	.08	0.12 (0.006-0.70)	.05
Switch to agree (n=54 responses)	0.70 (0.23-2.10)	.53	0.75 (0.17-3.29)	.70	4.54 (0.93-22.13)	.06

^a^PRIMO: Prime Implicant Maintenance of Outcome.

Table 3 presents the results of the GLMM fit to only responses on complex items, which were deemed likely to be difficult to classify. The data exhibited greater prior agreement on complex items in the PRIMO software group (total trials: 16/28, 57%) than for the monotonicity (11/28, 39%) and best-worst case (11/28, 39%) groups. However, Table 3 displays that despite the greater opportunity for participants to switch to disagree, for these items, the odds of participants in the PRIMO software group switching to disagree were 0.05 (95% CI 0.00-0.31) relative to the best-worst case group. Similarly, among the complex items for which there was prior disagreement, the odds of participants in the PRIMO software group switching incorrect prior responses to agree with ML models was over 4 times that of either of the other groups (odds ratio relative to the best-worst group 4.1; odds ratio relative to the monotonicity group 5.1).

**Figure 7:**
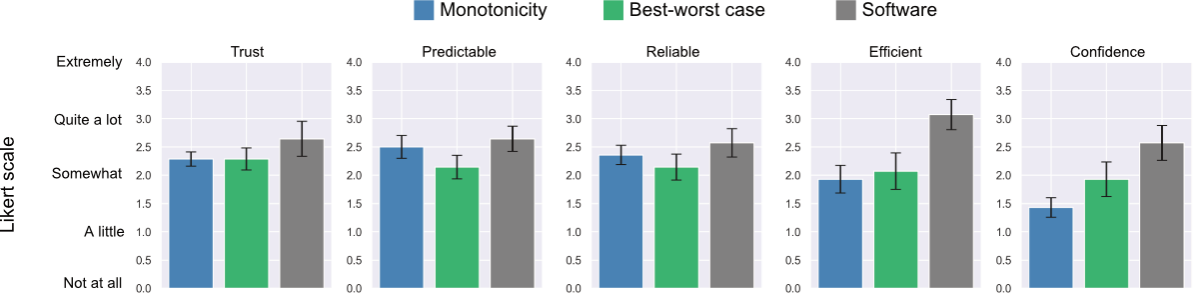
Likert scale analysis for intelligibility questionnaire (mean value and SE).

Figure 7 shows 5 plots, 1 for each question in the intelligibility questionnaire. Although there was no significant difference in intelligibility between the 3 explainability metrics (all *P*>.05), the result trended toward using the PRIMO software. Participants were more confident about their answers to the quantitative questions for the PRIMO software, followed by the best-worst case and monotonicity. On average, the participants found the PRIMO software to be more trustworthy, predictable, reliable, and efficient.

**Table 3:** Comparison between overall agree, switch to disagree, and switch to agree groups.

	Intercept, odds ratio (95% CI)	*P* value	Monotonicity, odds ratio (95% CI)	*P* value	PRIMO^a^ software, odds ratio (95% CI)	*P* value
Overall agree (complex)	0.714 (0.26-1.85)	.48	1.658 (0.53-5.42)	.39	8.056 (2.223-36.277)	.003
Switch to disagree (complex)	1.232 (0.0001-56,695.11)	.76	0.168 (0.00-1.00)	.16	0.05 (0.00-0.31)	.046
Switch to agreement (complex)	0.646 (0.14-2.71)	.52	0.814 (0.15-4.31)	.80	4.140 (0.705-35.64)	.14

^a^PRIMO: Prime Implicant Maintenance of Outcome.

### Qualitative Findings

#### Preference for Interactive Explanations

Participants found the PRIMO software to be the most understandable because of its ability to provide an explanation at an instance level. A few participants hoped to see explanations at the instance level while going beyond a global understanding of the model. Participant 2 stated, “Software was the most understandable and allowed for considering multiple individuals, multiple circumstances and really was user friendly even down to the level of a participant*.*” The ability of the participant to query and generate explanations that are of interest to them also played a role in their understanding of the model. A similar case was tested by Lakkaraju et al [51] who compared user interest-based subspace explanations with decision lists and decision sets.

#### Local Explanation Remains Insufficient for Answering the Question “Why?”

Many participants found the interactive nature of the PRIMO software and its ability to provide explanations to increase their understanding. However, a few participants were looking to reason from a behavioral perspective “why” the interpretable model generates ranges for the explanations the way it does. Participant 2 stated the following concerning the PRIMO software:

I really like the model. I would just say, somewhat predictable. I thought I was setting threshold at 3 weeks that would be likely associated with weight loss and was surprised that the model put the person in the not-losing weight category. I think it’s really good, really efficient, allows you to see the impact of your various inputs are.

Having gone through the details of the PRIMO software and using it a little, participant 11 stated the following:

So, if I wanted someone to understand it, I think you know your explanation was really interesting of this one because you said it tells you why, but it does not tell you why. It tells you what ranges are causing you to make that decision, but it’s not telling you why those ranges matter, for like the behavior.

It appears that explainability metrics alone are not sufficient to inform the user regarding the precise reasoning behind the outcome.

#### Missing Model Performance–Related Information

Previous literature demonstrates that showing a model’s accuracy on the held-out set can affect people’s perception of the model’s performance and their trust [52]. Therefore, throughout the study, domain experts were not shown any information about the performance of the RF model or the interpretable OBDD-based model, as our goal was to see how the explanation independently affects people’s trust in the ML model. However, participants were curious to know model performance metrics, especially while interacting with the PRIMO software:

[I] need to see metrics on how the model really performs in some automated fashion rather than me making changes to see. Human/human plus this software makes better predictions—would be the real test of this. In the sense, I can’t answer this because it’s not been evaluated objective.

Participant 4

I will still say quite a bit trustworthy. Because I’ll still like to see other overall model performance metrics about, I don’t know if accuracy would be the right one to use, but something like overall model performance would be nice to see as well, but I feel like I understand the model.

Participant 5

These model performance metrics provide an understanding of the overall model performance along with a global explanation. It seems that local or global explanations alone cannot fulfill the domain experts’ need to create mental models that describe the overall behavior of the model. Consequently, a combined global and local explanation approach is necessary, reinforcing prior findings in the explainability literature [24,51].

#### Features Related

The participants were shown results based on the 5 features that were used to train the RF model. These features were also selected based on permutation feature selection methods and guided by experts. However, a few participants, through their clinical and research practice, hoped to see features such as gender or calories. Having gone through the first local explanation type, participant 1 would have liked to have seen a lot more variables:

I would like a lot of variables; I wouldn’t stick just to these four. I don’t know how much I’d trust it just based on these five features.

Participant 7, based on their practice, felt that “The single most important one is calorie intake*.*”

Participant 6 also hoped to see a demographic feature and stated the following:

What’s interesting is that I wish that the participant’s sex was included as well because usually when I look at weight loss percentages I, evaluate them a little bit different for men vs women just because, in my clinical experience and from a research perspective, men tend to lose more weight than women.

Participant 13 thought it might be helpful to combine features, stating the following:

You’re saying that they’re [custom food item] separate from average food items, but what if you just add them together as one total variable.

#### Misinterpreting the Term Model

The term “model” refers to the ML model. In our case, it refers to the RF model that was trained and used to generate the interpretable model. A few participants confused the term model for the explanation type itself. Participant 3 was curious to know how the model weighted factors but intended to understand how the explanation was weighting different features:

I think somewhat [predictable] because I think when it comes to predict the individual participants, it was hard when not everything followed the principle of monotonicity so I could not decide how the model would end up weighting certain factors, so when things were inconsistent I was not sure how that would turn out.

Similarly, when asked if they trust the model guided by the explanation (they referred to trusting the model), participant 2, while going over the second explanation type, wanted to convey that they trusted the explanation:

Somewhat trust the model, because to a certain extent, it predicts and is consistent with what I know from literature. The fact that it seems to discount the completeness of the dietary record that makes me question it a little bit, but I do know it’s important to log fat, so I can see that being potentially important.

#### The Usefulness of Explanations to Patients

After having used the PRIMO software and testing it, participant 2 felt that it could be beneficial for patients to observe the thresholds determined by the explanations to understand their goal progress:

I can see even participants [patients] messing around with it and seeing what their goal thresholds might need to be. The same limitations in terms of binary categories and what I think is ideal.

Although an exciting hypothesis, we do not know how many patients would find such helpful information. Future research should explore such questions to determine whether providing PRIMO explanations would benefit patients.

#### Weight Loss Definition

A few participants were curious to know about the definition of weight loss itself. Specifically, what percentage of weight loss is clinically significant? Participant 13 expressed their views about a fixed threshold for defining weight loss and stated the following:

For example, if we were to categorize, like what weight change is right, is it, like what ’s the difference between someone who has 0.00 vs someone who has ‒0.01? But then one’s losing weight, and one’s not, and then that kind of like muddies the water in a way, so is it like that those who are in the middle of transitioning to something.” Several definitions of clinically significant weight loss vary based on the clinical outcome of interest. For example, weight loss as low as 2% has shown promise for preventing diabetes, whereas weight loss of 7% to 10% may be warranted to see changes in physical functioning.

## Discussion

### Principal Findings

We have demonstrated the feasibility of building a model that predicts weight loss as early as 2 weeks into an intervention using five engagement and diet-related features obtained from a smartphone app: (1) average daily food entry, (2) average daily custom food entry, (3) average weekly weight change, (4) average daily fat, and (5) average daily saturated fat. The model used yielded an 81% accuracy (specificity: 86% and sensitivity: 69%). Recent studies have demonstrated the predictive value of initial self-reported weight loss on long-term weight loss, which our model validates; however, most of these models achieve low specificity (eg, 50.5%-53.6% [53]), which demonstrates a challenge in predicting nonresponders. Predicting nonresponse early is essential to be able to change the course of treatment. We suggest that future predictive models assess engagement (eg, number of times participants log food and create custom food items) and self-reported fat intake (total fat and saturated fat) to advance their prediction of nonresponders. Although recent findings on engagement with smartphone apps (eg, interaction length and density of use [54]) have been shown to be predictive of positive health behavior trends, our findings support the use of specific features, such as daily entries of food logs and frequency of creation of new food items, as important predictors of weight loss in the context of weight loss treatment programs. Prior literature has shown support for fat intake positively predicting change in body fat [55], and our findings show that self-report based on food logging may be an important feature in the early prediction of nonresponse. This model is a first step toward saving intervention costs and improving health outcomes by using an effective stepped-care model. This saves intervention costs by predicting responders and potentially stepping down care and improves health outcomes by predicting nonresponders and stepping up care. Current studies, such as the SMART study [56], are beginning to test stepped-care approaches; however, they lack validated weight loss prediction models. Future work should further validate this model in a different study to assess its generalizability. We plan to analyze data from a completed study and assess whether RF models can help predict weight loss. We also plan to conduct a clinical trial in the future to study the predictive power of the 5 predictors we selected, in addition to day-to-day variability (Shahabi, F, unpublished data, September 2022), macronutrients, and the feasibility of using RF models and the PRIMO system as a decision support tool for stepped-care treatment.

We have also addressed one of the critical challenges of representing complex models using an explainability tool, PRIMO, an interactive tool that uses prime implicants as an explainability metric. Of the 14 weight management experts, 10 ranked the PRIMO software first in advancing their understanding of ML models and 3 ranked it second. Some participants felt that the PRIMO software was more reliable and user-friendly and allowed for greater engagement to consider different types of people. However, there was agreement that the PRIMO software system could still be improved by combining global explanations such as the accuracy of the model. Weight management experts desire to design a visualization tool that combines interactive instance-based explanations with interactive global explanations. Participants can then query the system to demonstrate the overall model performance (similar to the other 2 explainability metrics).

Another key challenge is whether weight management experts are more or less likely to use ML recommendations when provided with explainability metrics. In basic predictive scenarios, ML with explainability may be more powerful than other tools for encouraging weight management experts to switch to agree (when they make simple errors [57,58]). Although still powerful in switching to an agreement, in complex scenarios, ML with explainability is not more powerful than other simpler methods when switching the mind of a weight management expert when they disagree with the algorithm. However, it is more powerful than other basic tools when they agree with the algorithm to prevent them from changing their minds (ie, switching to disagree), acting as a great confirmatory tool. When looking at switching to agree, the PRIMO software tool had high numbers in both the basic and complex scenarios, and in complex scenarios, it showed to be significantly more likely to agree after viewing the PRIMO software. Future work should further investigate the effect of ML with explainability tools when the predictions are considered less controversial (basic) compared with complex scenarios.

Despite the increased potential for the adoption of ML models with explainability metrics, it is unclear whether an interactive PRIMO software that uses ML models with explainability would significantly advance trust compared with other methods. We notice that PRIMO software, on average, yields the greatest trust (mean 2.64, SD 1.15 vs 2.29, SD 0.47 for monotonicity and 2.29, SD 0.73 for the best-worst case); however, it is not significantly different from other methods. Most participants liked the PRIMO software because of its interactive nature and felt it was more transparent and “trustworthy” than the other 2 methods. However, many still felt that although they could test different hypothetical individuals and see the impact of these changes on model output, they still did not fully understand how the PRIMO software was generating these predictions and windows of uncertainty. They liked that they could see the combination of the different features and what they were likely to predict; however, it seems that with any local explanation, people still wanted to see the overall metrics (eg, accuracy and specificity or sensitivity).

### Limitations

Despite following the principles of designing human explanations, there remain challenges in understanding these models. Our findings support that the understanding of ML models is improved through explainability metrics. However, our interactive PRIMO software method did not show significant improvements over other explainability methods, especially in terms of efficiency, predictability, and confidence. In explainability metrics, we must recognize that we are still only learning a functional relationship between a set of features (ie, consumption of fat or entry of a food item into the app) and a class label (yes or no to weight loss after 6 months of intervention), uncovering the patterns the RF finds in the data. However, as 1 participant mentioned, to truly uncover an understanding, we must answer the “Why?” which requires building models of understanding the causal mechanisms (including the latent ones), as discussed by Pearl and Mackenzie [59] and Darwiche [60]. Although researchers often refer to the output of a classifier trained on data as a learned model, in reality, it is a learned function. It has become increasingly apparent that the ability to answer the “Why?” question depends on whether one is learning a *function* or a causal *model*. For example, we learn a functional relationship between a feature vector and a class label by learning a classifier using RF from the data. Hence, a local explanation may only provide insight into the sort of pattern an RF algorithm found in the data. In contrast, with a *model*, we try to represent the underlying causal mechanisms of a system (including latent ones); therefore, we may be able to answer questions such as, “Why does consuming more fat increase the odds of gaining weight?” In other words, although local explanations may help increase one’s trust in a classifier that was learned from data, the ability to answer “Why?” questions may require that we go beyond functions and classifiers and instead seek to learn and develop causal models [59,60]. Our findings show promise in PRIMO gaining the trust of weight management experts; however, the lack of significance compared with other methods may be attributed to our relatively small sample size (n=14) and to the fact that defining trust in ML models or functions remains elusive.

### Conclusions and Future Work

We performed a qualitative analysis of the one-on-one guided interviews with weight management experts to study whether an interactive explainability tool can enhance trust and understanding of the behavior of the original model, thereby increasing adoption. Our findings show that domain experts showed a significant increase in agreement when using our interactive explainability tool compared with other methods, especially when it comes to explaining basic weight loss archetypes (people that are easier to predict). This shows the potential for interactive explainability tools to catch errors or misjudgments in weight management experts’ initial predictions. Moreover, we showed that participants were significantly less likely to change their responses to disagree when their initial predictions were aligned with the interactive prediction tool [61]. In general, when shown local explanations of specific hypothetical archetypes, participants felt the need to understand the model’s behavior at a global level. Alternatively, when shown global explanations, participants felt the need to understand how specific instances would be classified and explained by the model. Therefore, augmenting our interactive explainability tool to include a visual representation combining local and global explanations could further enhance the agreement with ML models. In addition, model performance metrics, such as accuracy, sensitivity, and specificity, are also needed to enable domain experts to gauge their reliability in the ML model’s decisions to avoid overreliance. This implies that explanations are required at every step in the framework, from visualizing and understanding trends in raw data to using the model to make predictions. Through our findings, we also learn that domain experts often differ, when compared with data scientists, in the use of the term “model” where data scientists often conflate learned models with learned functions. Aside from a more refined use of terminology, expectations and limitations need to be set in terms of what can be gained from a learned function compared with a learned model. Future studies could include testing a more comprehensive explanation interface with aspects of local and global explanations. Weight management interventions may benefit from such explanations by testing the delivery of just-in-time adaptive interventions based on the explanation ranges generated by the PRIMO software to change the course of treatment. The just-in-time adaptive intervention could explain to the patients in the treatment program why the system thinks they need to stay or change a course of treatment, potentially increasing users’ trust in the system.

This work provides a foundation to aid in translating models in communicating with other disciplines such that their use in research contexts is more plausible. To the extent that researchers can trust, use, and explain models to guide decisions, these could be used in research protocols to guide testable treatment decisions based on ML, rather than less-complex or less-accurate correlational or clinical intuition methods. Future studies should also test the effectiveness of these explanations among patients and clinicians. We plan to provide PRIMO as an open-source software tool for the community so that not only weight management researchers but also computer science researchers can build on this framework to develop more rigorous tools for clinical decision support.

## Appendix

Multimedia Appendix 1 Summary video.

Multimedia Appendix 2 Feature details.

Multimedia Appendix 3 Participant demographics (interviewees).

Multimedia Appendix 4 Visuals of the explainability metrics shown to participants for monotonicity, PRIMO (Prime Implicant Maintenance of Outcome) software, and best-worst case analysis.

## Abbreviations

AI: artificial intelligence
GLMM: generalized linear mixed-effects model
LIME: local interpretable model-agnostic explanations
LRT: likelihood ratio test
ML: machine learning
OBDD: ordered binary decision diagrams
PRIMO: Prime Implicant Maintenance of Outcome
REDCap: Research Electronic Data Capture
RF: random forest
SHAP: Shapley additive explanations

## References

[1] Biener A, Cawley J, Meyerhoefer C (2017). The high and rising costs of obesity to the US health care system. J Gen Intern Med.

[2] Cawley J, Biener A, Meyerhoefer C, Ding Y, Zvenyach T, Smolarz BG, Ramasamy A (2021). Direct medical costs of obesity in the United States and the most populous states. J Manag Care Spec Pharm.

[3] Islam MM, Poly TN, Walther BA, Jack Li YC (2020). Use of mobile phone app interventions to promote weight loss: meta-analysis. JMIR Mhealth Uhealth.

[4] Cai X, Qiu S, Luo D, Wang L, Lu Y, Li M (2020). Mobile application interventions and weight loss in type 2 diabetes: a meta-analysis. Obesity (Silver Spring).

[5] Farage G, Simmons C, Kocak M, Klesges RC, Talcott GW, Richey P, Hare M, Johnson KC, Sen S, Krukowski R (2021). Assessing the contribution of self-monitoring through a commercial weight loss app: mediation and predictive modeling study. JMIR Mhealth Uhealth.

[6] Bower P, Gilbody S (2005). Stepped care in psychological therapies: access, effectiveness and efficiency. Narrative literature review. Br J Psychiatry.

[7] Finkelstein J, Jeong IC (2017). Machine learning approaches to personalize early prediction of asthma exacerbations. Ann N Y Acad Sci.

[8] Tripathi G, Kumar R (2020). Early prediction of diabetes mellitus using machine learning. Proceedings of the 8th International Conference on Reliability, Infocom Technologies and Optimization (Trends and Future Directions).

[9] Martinez DA, Levin SR, Klein EY, Parikh CR, Menez S, Taylor RA, Hinson JS (2020). Early prediction of acute kidney injury in the emergency department with machine-learning methods applied to electronic health record data. Ann Emerg Med.

[10] Moor M, Rieck B, Horn M, Jutzeler CR, Borgwardt K (2021). Early prediction of sepsis in the ICU using machine learning: a systematic review. Front Med (Lausanne).

[11] Opoku Asare K, Terhorst Y, Vega J, Peltonen E, Lagerspetz E, Ferreira D (2021). Predicting depression from smartphone behavioral markers using machine learning methods, hyperparameter optimization, and feature importance analysis: exploratory study. JMIR Mhealth Uhealth.

[12] Kim M, Yang J, Ahn WY, Choi HJ (2021). Machine learning analysis to identify digital behavioral phenotypes for engagement and health outcome efficacy of an mHealth intervention for obesity: randomized controlled trial. J Med Internet Res.

[13] Akour I, Alshurideh M, Al Kurdi B, Al Ali A, Salloum S (2021). Using machine learning algorithms to predict people's intention to use mobile learning platforms during the COVID-19 pandemic: machine learning approach. JMIR Med Educ.

[14] Yu J, Chiu C, Wang Y, Dzubur E, Lu W, Hoffman J (2021). A machine learning approach to passively informed prediction of mental health risk in people with diabetes: retrospective case-control analysis. J Med Internet Res.

[15] Mori T, Uchihira N (2018). Balancing the trade-off between accuracy and interpretability in software defect prediction. Empir Softw Eng.

[16] Yoo J, Kim SH, Hur S, Ha J, Huh K, Cha WC (2021). Candidemia risk prediction (CanDETEC) model for patients with malignancy: model development and validation in a single-center retrospective study. JMIR Med Inform.

[17] Surodina S, Lam C, Grbich S, Milne-Ives M, van Velthoven M, Meinert E (2021). Machine learning for risk group identification and user data collection in a herpes simplex virus patient registry: algorithm development and validation study. JMIRx Med.

[18] O'Driscoll R, Turicchi J, Hopkins M, Duarte C, Horgan GW, Finlayson G, Stubbs RJ (2021). Comparison of the validity and generalizability of machine learning algorithms for the prediction of energy expenditure: validation study. JMIR Mhealth Uhealth.

[19] Cacheda F, Fernandez D, Novoa FJ, Carneiro V (2019). Early detection of depression: social network analysis and random forest techniques. J Med Internet Res.

[20] Chatzimparmpas A, Martins RM, Jusufi I, Kucher K, Rossi F, Kerren A (2020). The state of the art in enhancing trust in machine learning models with the use of visualizations. Comput Graph Forum.

[21] Henry K, Kornfield R, Sridharan A, Linton RC, Groh C, Wang T, Wu A, Mutlu B, Saria S (2022). Human-machine teaming is key to AI adoption: clinicians' experiences with a deployed machine learning system. NPJ Digit Med.

[22] Bussone A, Stumpf S, O'Sullivan D (2015). The role of explanations on trust and reliance in clinical decision support systems. Proceedings of the 2015 International Conference on Healthcare Informatics.

[23] Jacobs M, Pradier MF, McCoy Jr TH, Perlis RH, Doshi-Velez F, Gajos KZ (2021). How machine-learning recommendations influence clinician treatment selections: the example of the antidepressant selection. Transl Psychiatry.

[24] Lundberg SM, Lee SI (2017). A unified approach to interpreting model predictions. Proceedings of the 31st International Conference on Neural Information Processing Systems.

[25] Ribeiro M, Singh S, Guestrin C (2016). “Why should I trust you?”: Explaining the predictions of any classifier. Proceedings of the 2016 Conference of the North American Chapter of the Association for Computational Linguistics: Demonstrations.

[26] Ribeiro MT, Singh S, Guestrin CE (2018). Anchors: high-precision model-agnostic explanations. Proceedings of the Thirty-Second AAAI Conference on Artificial Intelligence and Thirtieth Innovative Applications of Artificial Intelligence Conference and Eighth AAAI Symposium on Educational Advances in Artificial Intelligence.

[27] Mohseni S, Zarei N, Ragan ED (2021). A multidisciplinary survey and framework for design and evaluation of explainable AI systems. ACM Trans Interact Intell Syst.

[28] Alvarez Melis D, Kaur H, Daumé III H, Wallach H, Wortman Vaughan JW (2021). From human explanation to model interpretability: a framework based on weight of evidence. HCOMP '21.

[29] Shih AY, Choi AY, Darwiche A (2019). Compiling Bayesian Network Classifiers into Decision Graphs. Proceedings of the 33rd AAAI Conference on Artificial Intelligence and 31st Innovative Applications of Artificial Intelligence Conference and 9th AAAI Symposium on Educational Advances in Artificial Intelligence.

[30] Shi W, Shih A, Darwiche A, Choi A (2020). On tractable representations of binary neural networks. Proceedings of the 17th International Conference on Principles of Knowledge Representation and Reasoning.

[31] Choi A, Shih A, Goyanka A, Darwiche A On symbolically encoding the behavior of random forests. arXiv..

[32] Kim E, Park YB, Choi K, Lim YW, Ok JM, Noh EY, Song TM, Kang J, Lee H, Kim SY (2020). Application of machine learning to predict weight loss in overweight, and obese patients on Korean medicine weight management program. J Korean Med.

[33] Pellegrini CA, Hoffman SA, Collins LM, Spring B (2014). Optimization of remotely delivered intensive lifestyle treatment for obesity using the multiphase optimization strategy: Opt-IN study protocol. Contemp Clin Trials.

[34] Spring B, Pfammatter AF, Marchese SH, Stump T, Pellegrini C, McFadden HG, Hedeker D, Siddique J, Jordan N, Collins LM (2020). A factorial experiment to optimize remotely delivered behavioral treatment for obesity: results of the Opt-IN study. Obesity (Silver Spring).

[35] Knowler WC, Barrett-Connor E, Fowler SE, Hamman RF, Lachin JM, Walker EA, Nathan DM, Diabetes Prevention Program Research Group (2002). Reduction in the incidence of type 2 diabetes with lifestyle intervention or metformin. N Engl J Med.

[36] Patel ML, Hopkins CM, Bennett GG (2019). Early weight loss in a standalone mHealth intervention predicting treatment success. Obes Sci Pract.

[37] Pfammatter AF, Nahum-Shani I, DeZelar M, Scanlan L, McFadden HG, Siddique J, Hedeker D, Spring B (2019). SMART: study protocol for a sequential multiple assignment randomized controlled trial to optimize weight loss management. Contemp Clin Trials.

[38] Waring ME, Schneider KL, Appelhans BM, Busch AM, Whited MC, Rodrigues S, Lemon SC, Pagoto SL (2014). Early-treatment weight loss predicts 6-month weight loss in women with obesity and depression: implications for stepped care. J Psychosom Res.

[39] Hooper L, Abdelhamid A, Moore HJ, Douthwaite W, Skeaff CM, Summerbell CD (2012). Effect of reducing total fat intake on body weight: systematic review and meta-analysis of randomised controlled trials and cohort studies. BMJ.

[40] Champagne CM, Broyles ST, Moran LD, Cash KC, Levy EJ, Lin PH, Batch BC, Lien LF, Funk KL, Dalcin A, Loria C, Myers VH (2011). Dietary intakes associated with successful weight loss and maintenance during the weight loss maintenance trial. J Am Diet Assoc.

[41] Lin P, Grambow S, Intille S, Gallis JA, Lazenka T, Bosworth H, Voils CL, Bennett GG, Batch B, Allen J, Corsino L, Tyson C, Svetkey L (2018). The association between engagement and weight loss through personal coaching and cell phone interventions in young adults: randomized controlled trial. JMIR Mhealth Uhealth.

[42] Carey A, Yang Q, DeLuca L, Toro-Ramos T, Kim Y, Michaelides A (2021). The relationship between weight loss outcomes and engagement in a mobile behavioral change intervention: retrospective analysis. JMIR Mhealth Uhealth.

[43] Darwiche A, Marquis P (2002). A knowledge compilation map. J Artif Intell Res.

[44] Shih A RF_SDD. GitHub.

[45] Shih A, Choi A, Darwiche A (2018). A symbolic approach to explaining Bayesian network classifiers. Proceedings of the 27th International Joint Conference on Artificial Intelligence.

[46] Shih A, Choi A, Darwiche A (2018). Formal verification of Bayesian network classifiers. Proc Mach Learn Res.

[47] Darwiche A, Hirth A (2020). On the reasons behind decisions. Proceedings of the 24th European Conference on Artificial Intelligence.

[48] Coudert O, Madre JC, Fraisse H, Touati H (1993). Implicit prime cover computation: An overview. Proceedings of the 1993 Synthesis And SImulation Meeting and International interchange.

[49] Coudert O, Madre JC (1993). Fault tree analysis: 10/sup 20/ prime implicants and beyond. Proceedings of the 1993 Annual Reliability and Maintainability Symposium.

[50] Cahour B, Forzy JF (2009). Does projection into use improve trust and exploration? An example with a cruise control system. Saf Sci.

[51] Lakkaraju H, Kamar ES, Caruana RA, Leskovec J (2019). Faithful and customizable explanations of black box models. Proceedings of the 2019 AAAI/ACM Conference on AI, Ethics, and Society.

[52] Yin M, Wortman JW, Wallach HM (2019). Understanding the effect of accuracy on trust in machine learning models. Proceedings of the 2019 CHI Conference on Human Factors in Computing Systems.

[53] Al-Abdullah L, Welsh P, Logue J (2022). Development of a predictive model for shortmedium-term weight loss in people with type 2 diabetes attending a weight management programme. Diabetologia.

[54] Alshurafa N, Jain J, Alharbi R, Iakovlev G, Spring B, Pfammatter A (2018). Is more always better?: Discovering incentivized mHealth intervention engagement related to health behavior trends. Proc ACM Interact Mob Wearable Ubiquitous Technol.

[55] Särnblad S, Ekelund U, Aman Jan (2006). Dietary fat intake predicts 1-year change in body fat in adolescent girls with type 1 diabetes. Diabetes Care.

[56] Grilo C, White M, Masheb R, Ivezaj V, Morgan P, Gueorguieva R (2020). Randomized controlled trial testing the effectiveness of adaptive "SMART" stepped-care treatment for adults with binge-eating disorder comorbid with obesity. Am Psychol.

[57] Eom JH, Kim SC, Zhand BT (2008). AptaCDSS-E: a classifier ensemble-based clinical decision support system for cardiovascular disease level prediction. Expert Syst Appl.

[58] Antoniadi AM, Du Y, Guendouz Y, Wei L, Mazo C, Becker BA, Mooney C (2021). Current challenges and future opportunities for XAI in machine learning-based clinical decision support systems: a systematic review. Appl Sci.

[59] Pearl J, Mackenzie D (2018). The Book of Why: The New Science of Cause and Effect.

[60] Darwiche A (2018). Human-level intelligence or animal-like abilities?. Commun ACM.

[61] Diprose WK, Buist N, Hua N, Thurier Q, Shand G, Robinson R (2020). Physician understanding, explainability, and trust in a hypothetical machine learning risk calculator. J Am Med Inform Assoc.

