# Supplementary figures and images for: An Explainable Artificial Intelligence Software Tool for Weight Management Experts (PRIMO): Mixed Methods Study
Source: J Med Internet Res. 2023 Sep 6;25:e42047. doi: 10.2196/42047 (PMC10512114; doi:10.2196/42047)

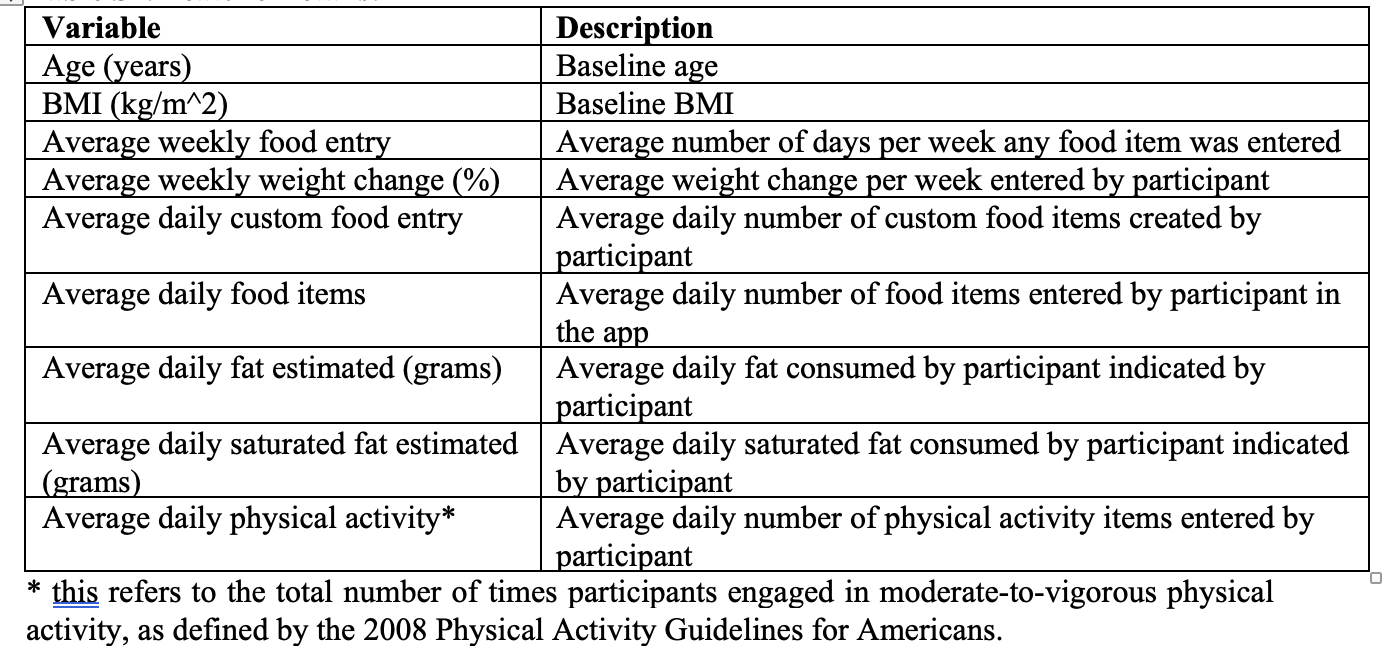

Supplement: Multimedia Appendix 2 [file jmir_v25i1e42047_app2.png]

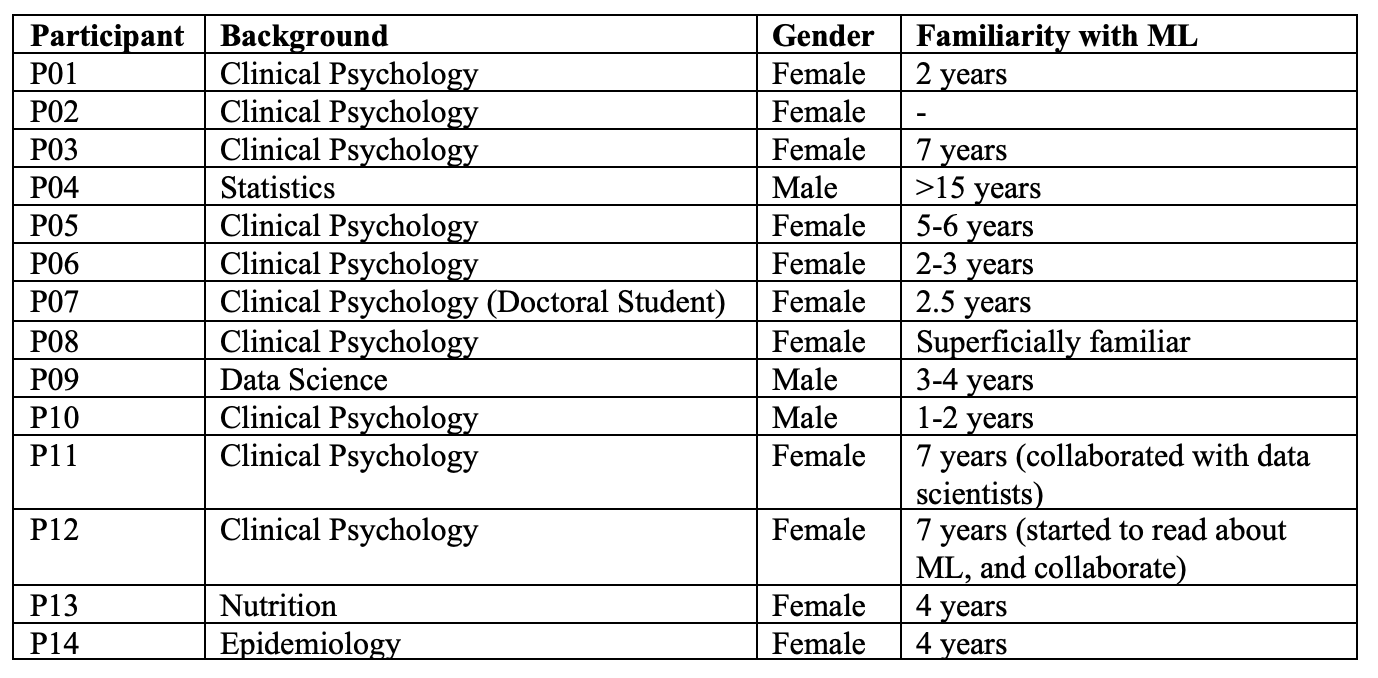

Supplement: Multimedia Appendix 3 [file jmir_v25i1e42047_app3.png]

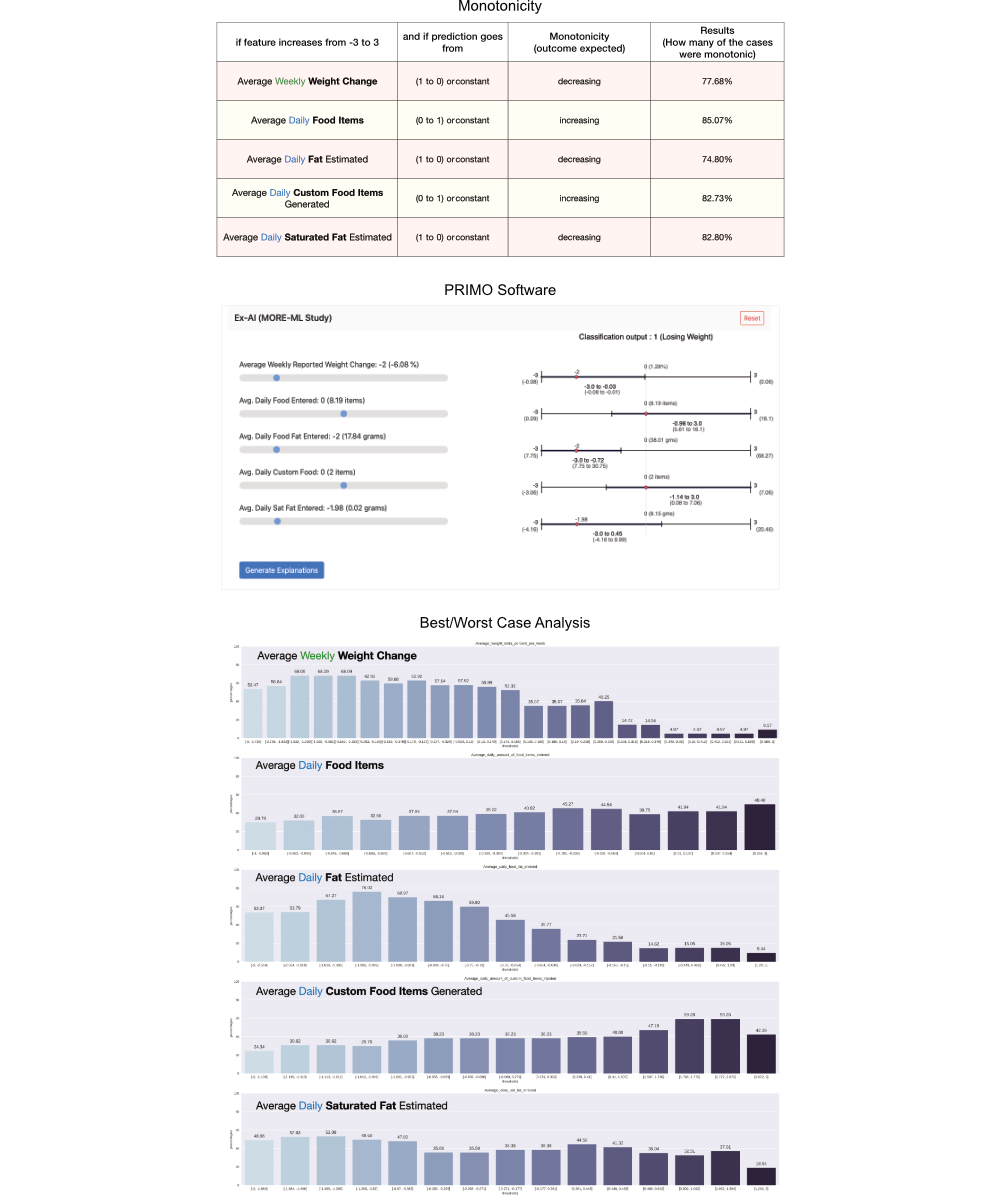

Supplement: Multimedia Appendix 4 [file jmir_v25i1e42047_app4.png]
